# EUS-HGS with antegrade stenting vs. hepaticogastrostomy alone for malignant biliary drainage: Systematic review and meta-analysis

**DOI:** 10.1055/a-2840-6762

**Published:** 2026-04-10

**Authors:** Tawfik Khoury, Wisam Sbeit, Fabien Fumex, Pietro Fusaroli, Graziella Masciangelo, Angelo Bruni, Giovanni Barbara, Andrea Anderloni, Masayuki Kitano, Masahiro Itonaga, Takeshi Ogura, Carlos A. Praticò, Rodica Gincul, Sarah Leblanc, Anthony Y.B. Teoh, Jeremie Jacques, Bertrand Napoleon, Andrea Lisotti

**Affiliations:** 1 61255 Department of Gastroenterology, Galilee Medical Center, Nahariya, Israel; 2 26731 Azrieli Faculty of Medicine, Bar-Ilan University, Ramat Gan, Israel; 3 61255 Gastroenterology, Nahariya Western Galilee Hospital, Nahariya, Israel; 4 89686 Endoscopy Unit, Hôpital Privé Jean Mermoz, Lyon, France; 5 9296 Gastroenterology Unit, Hospital of Imola, University of Bologna, Imola, Italy; 6 198207 Gastroenterology Unit, Department of Medical and Surgical Sciences, University of Bologna, Bologna, Italy; 7 CEI Centre Endoscopie Interventionnelle, Hôpital Privé Armand Brillard, Nogent-sur-Marne, France; 8 9268 Digestive Endoscopy Unit, Division of Gastroenterology, IRCCS Humanitas Research Hospital, Rozzano, Italy; 9 175768 Second Department of Internal Medicine, Wakayama Medical University School of Medicine, Graduate School of Medicine, Wakayama, Japan; 10 13010 2nd Department of Internal Medicine, Osaka Medical College, Takatsuki, Japan; 11 13620 Surgery, Hong Kong Sanatorium & Hospital Limited, Hong Kong, Hong Kong; 12 558112 service d'hépato-gastro-entérologie, University Hospital Centre of Limoges Dupuytren 2, Limoges, France

**Keywords:** Pancreatobiliary (ERCP/PTCD), Strictures, Endoscopic ultrasonography, Biliary tract, Intervention EUS, Quality and logistical aspects, Performance and complications

## Abstract

**Background and study aims:**

Endoscopic ultrasound-guided hepaticogastrostomy (EUS-HGS) is an effective and safe therapeutic option for biliary drainage in patients with malignant biliary obstruction (MBO). Several authors proposed use of antegrade stenting (AS) combined with EUS-HGS to improve long-term outcomes, with controversial results. We aimed to assess pooled performance of EUS-HGS+AS compared with EUS-HGS alone.

**Methods:**

Database search was performed to identify studies comparing EUS-HGS+AS to EUS-HGS alone for biliary drainage in patients with MBO. Primary outcome was recurrent biliary obstruction (RBO). Secondary outcomes were technical, clinical success, adverse events (AEs), severe AEs rate, time to RBO, and overall survival (OS). Relative risks (RRs) with 95% confidence intervals (CIs) were calculated using random-effect model.

**Results:**

Five studies involving 555 patients were retrieved. RBO was lower in patients who underwent EUS-HGS+AS (RR 0.30; [0.18–0.49]; *P* < 0.001). Pooled technical success, clinical success, AE, and severe AE rates were similar (RR 0.94 [0.85–1.05], RR 1.02 [0.94–1.11], RR 0.88 [0.50–1.55]), and 0.26 [0.03–2.22], respectively). Time to RBO was higher in EUS-HGS+AS (SMD + 4.02 [0.57–7.47]; *P* = 0.04). Mean procedure time was similar among the groups (SMD +0.38 [-0.12–0.87]; *P* = 0.13) as well as OS was similar in the two groups (SMD 0.18 [-0.20–0.52]; *P* = 0.85).

**Conclusions:**

Combining AS with EUS-HGS reduces RBO risk in patients with MBO, without impact on technical, clinical success rates, or safety profile. Randomized controlled trials are needed to confirm these observations.

## Graphical Abstract

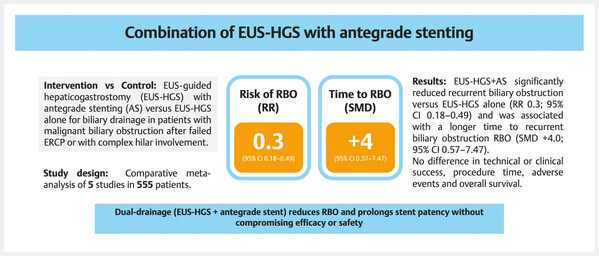

## Introduction

Endoscopic retrograde cholangiopancreatography (ERCP) is currently considered the first-line approach for treatment of malignant biliary obstruction (MBO). In case of ERCP failure, mainly due to unsuccessful cannulation or unreachable papilla, endoscopic ultrasound (EUS) biliary drainage is considered the preferred treatment strategy in terms of clinical outcomes and safety profile 1 2 . To date, EUS-hepaticogastrostomy (EUS-HGS) is recommended in patients suffering from MBO after failed ERCP or in case of complex hilar strictures, requiring multimodal approaches (endoscopic, EUS or percutaneous) 3 4 .

Previous meta-analysis reported a pooled technical and clinical success for EUS-HGS of 96% and 90%, respectively 5 . Pooled incidence of adverse events (AEs) was 5.8%, 12.1%, 4.2%, and 3.7% for mild, moderate, severe, and fatal AEs, respectively; bile leak, stent occlusion, stent migration, and cholangitis represent the most frequent AEs observed after EUS-HGS 6 .

In the last decade, a slow but progressive improvement in treatment outcomes and survival in patients suffering from MBO receiving chemotherapy was observed; thus, incidence of recurrent biliary obstruction (RBO) could dramatically impact on the clinical course of the disease 7 8 . A recent meta-analysis reported that patients undergoing EUS-HGS for MBO report a pooled 16.2% incidence of RBO after a median of 165 days 9 .

Recently, several studies explored the potential effect of combining antegrade stenting (AS) to EUS-HGS on the main clinical outcomes of biliary drainage, reporting inhomogeneous results 10 11 . Two meta-analyses including all studies assessing outcomes of EUS-HGS and EUS-HGS combined to AS (EUS-HGS+AS) demonstrated that the combined transmural and antegrade approach to MBO leads to a reduction in need for reintervention due to RBO; these two meta-analyses included also non-comparative studies assessing the two treatment arms 12 13 . Thus, the aim of our study was to assess the pooled performance of EUS-HGS+AS compared to EUS-HGS alone for biliary drainage in patients suffering from MBO, including only comparative studies. The primary outcome was RBO rate, whereas technical and clinical success rates, procedure time, incidence and severity of AEs, time to RBO, and overall survival were secondary outcomes.

## Methods

This systematic review with meta-analysis was conducted according to the PRISMA guidelines. 14 . This systematic review was not prospectively registered in an international database. Ethics committee approval was not required as this study was based exclusively on previously published data.

### Selection criteria

Studies included in this meta-analysis were original comparative studies that met the following inclusion criteria: 1) Patients: adult patients with MBO; 2) Intervention: EUS-HGS combined with AS; 3) Comparator: EUS-HGS alone; and 4) Outcomes: primary outcome was RBO, whereas secondary outcomes were technical and clinical success, incidence of AEs, incidence of severe AEs, mean time to RBO, and mean overall survival. We excluded: 1) studies conducted with other design than comparative studies; 2) studies not reporting the primary outcome; 3) studies reporting outcomes of other approaches for biliary drainage than EUS-HGS with or without AS; and 4) studies enrolling patients undergoing EUS-HGS for benign biliary obstruction.

### Search strategy

A PubMed/Medline, Embase and Cochrane databases bibliographic research was conducted and limited to English language until the end of June 2025, independently by two authors (TK and AL) using the following search string: “("EUS" OR "EUS-guided" OR "Endosonography"[Mesh] OR "Endoscopic ultrasound") AND (“hepaticogastrostomy” OR “EUS-HGS”)”. An additional database search using Google Scholar and by checking the reference list of all relevant studies on this topic was conducted. In cases of overlap publications from the same population, the most recent reference was included.

### Quality assessment

Each study was evaluated and classified by two independent investigators (WS and GM). Discrepancies among reviewers about qualitative and quantitative data collection were solved through discussion and, if necessary, arbitration by a third reviewer (PF). Risk of bias for the included retrospective comparative studies was evaluated using the Risk Of Bias In Non‑randomized Studies-of Interventions (ROBINS‑I) tool, Version 2, which is designed to assess the certainty of effect estimates from non‑randomized intervention studies by examining key bias domains. For each study and outcome, seven domains were systematically assessed: bias due to confounding, bias in selection of participants into the study, bias in classification of interventions, bias due to deviations from intended interventions, bias due to missing data, bias in measurement of outcomes, and bias in selection of the reported result. Signaling questions within each domain guided judgements, which were then mapped to standard risk‑of‑bias categories (low, moderate, serious, critical) for each domain and overall. [ https://methods.cochrane.org/bias/risk-bias-non-randomized-studies-interventions ]

### Data extraction

The following data were collected for each included study: first author’s name; year of publication; journal of publication, study population, location of MBO, cause of MBO, presence of ascites, type of the stent used, technical success, clinical success, RBO, time to RBO, overall survival, incidence, and severity of AEs. In case of missing data, the corresponding authors have been contacted by email. In case of missing data for time to RBO or overall survival, the Kaplan-Meier curves of each study have been assessed and used for estimation with the use of the software package metaSurv for R available at https://www.divat.fr/en/softwares/metasurv and downloaded on November 1, 2024.

Two studies by Ishiwatari et al. were included. 15 16 The respective corresponding authors have been contacted to exclude any overlap between the two study populations. The corresponding authors stated that no population overlap is present. Moreover, the later study represented a propensity score-matched (PSM) analysis with different inclusion criteria and adjusted comparative estimates. Both studies, therefore, were retained because they address the research question using distinct analytical approaches. 15 16 Following the first peer-review round, all stent-related variables were independently re-verified against the original publications to ensure data accuracy.

### Outcomes definitions

Primary outcome was RBO defined as defined as occurrence of cholangitis or jaundice coupled with biliary dilation on imaging modalities. Secondary outcome were: 1) technical success defined as complete and accurate deployment of both stents in the EUS-HGS + AS group or the hepaticogastrostomy in the EUS-HGS alone group; 2) clinical success defined as improvement of cholangitis after biliary drainage and/or a decrease in serum total bilirubin by 50 % or a decrease to < 3 m/dL within 2 weeks; 3) occurrence of AEs (graded according to the American Society of Gastrointestinal Endoscopy lexicon); 4) time to RBO defined as the period from biliary drainage to RBO occurrence; and 5) overall survival.

### Statistical analysis

Pooled performance of EUS-HGS+AS and EUS-HGS alone were assessed and analyzed using a DerSimonian and Laird model; given the high degree of heterogeneity, random-effects models were applied for all pooled analyses. Dichotomous variables were reported as relative risk (RR) with 95% confidence interval (CI); continuous variables were reported as mean difference. Heterogeneity was assessed through I² tests. Publication bias was assessed through visual assessment of funnel plots and using the Egger’s test. The following sensitivity analyses were performed: 1) study population > 80 patients; 2) single-center vs. multicenter studies; 3) retrospective vs. PSM studies; 4) publication year (before and after 2023); 5) type of stent used for AS (metal vs. plastic); and 6) location of MBO (only distal MBO vs. both proximal and distal MBO). Statistical analysis was performed with MedCalc Statistical Software version 23.0.6 (MedCalc Software Ltd, Ostend, Belgium; https://www.medcalc.org ; 2024).

## Results

### Literature search and study characteristics

Electronic database search has been reported according to PRISMA guidelines in **Supplementary Fig. 1** . The literature search yielded 709 studies. After removal of duplication and preliminary screening of titles and abstract 30 publications were fully reviewed. Finally, five comparative studies were finally included in this meta-analysis 15 16 17 18 19 . Table 1 summarizes studies characteristics.

**Fig. 1:**
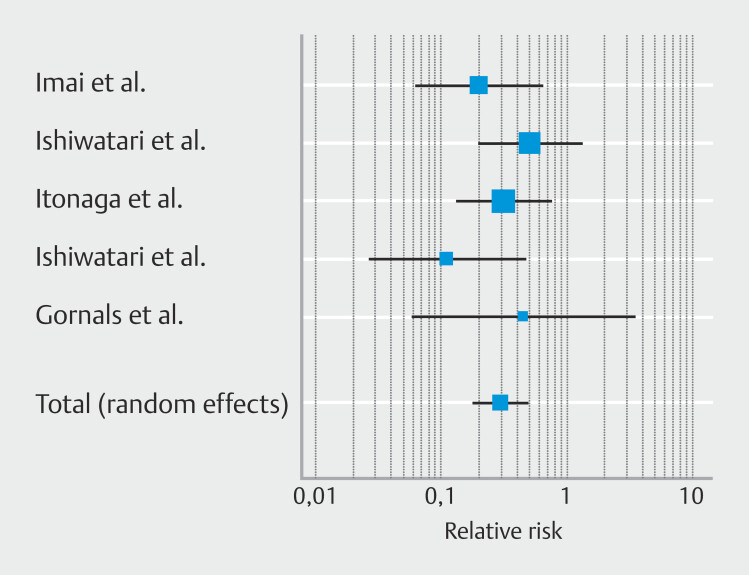
Forest plot reporting pooled estimates for recurrent biliary obstruction.

**Table:** **Table 1** Baseline study characteristics.

	Gornals et al.	Ishiwatari et al.	Itonaga et al.	Ishiwatari et al.	Imai et al.
Year of publication	2025	2024	2024	2022	2017
Journal	Endoscopy ABS (ESGE Days)	Gastrointestinal endoscopy	Int J Clin Oncol	J Hepatobiliary Pancreat Sci	Oncology
Study design	Propensity score matched	Propensity score matched	Retrospective	Retrospective	Retrospective
Patients number in EUS-HGS+AS group	31	81	30	38	37
Patients number in EUS-HGS group	125	81	32	58	42
**Cause of MBO in EUS-HGS+AS group (%)**					
Pancreatic cancer	NR	54.3	58	60.5	24.3
Cholangiocarcinoma	NR	4.9	17	2.6	29.7
Gastric cancer	NR	24.7	20	-	-
Duodenal cancer	NR	1.2	-	-	-
Others	NR	14.8	7	36.8	45.9
**Cause of MBO in EUS-HGS group (%)**					
Pancreatic cancer	NR	49.4	59	53.4	31
Cholangiocarcinoma	NR	3.7	34	12.1	42.9
Gastric cancer	NR	27.2	3	-	-
Duodenal cancer	NR	3.7	-	-	-
Others	NR	16	3	34.5	26.2
**Ascites (%)**					
EUS-HGS+AS group	NR	34.6	27	18.4	NR
EUS-HGS group	NR	32.1	13	17.2	NR
**Technical success (%)**					
EUS-HGS+AS group	100%	96.3	80	100	83.8
EUS-HGS group	96%	96.3	100	100	97.6
**Clinical success (%)**					
EUS-HGS+AS group	96%	NR	100	97.3	90.3
EUS-HGS group	79%	NR	92	94.8	90.2
**Puncture site at B2 (%)**					
EUS-HGS+AS group	NR	23.5	13	50	NR
EUS-HGS group	NR	27.2	13	36.2	NR
**Puncture site at B3 (%)**					
EUS-HGS+AS group	NR	76.5	87	50	NR
EUS-HGS group	NR	69.1	87	63.8	NR
**Stent used for AS in EUS-HGS+AS group (%)**					
Covered metallic	NR	24.4	0	0	00
Uncovered metallic	NR	75.6	0	100	100
Plastic	NR	0	75	0	00
Plastic + temporary naso-biliary tube	NR	0	25	0	00
**Stent used for EUS-HGS in EUS-HGS+AS group (%)**					
Metallic	NR	86.4	100	60.5	100
Plastic	NR	13.6	0	39.5	0
**Stent used for EUS-HGS in EUS-HGS group (%)**					
Metallic	NR	100	100	89.7	100
Plastic	NR	0	0	10.3	0
AS, antegrade stenting; EUS-HGS, endoscopic ultrasound hepaticogastrostomy; MBO, malignant biliary obstruction; NR, not reported.					

### Quality assessment

Risk of bias assessment evaluated using ROBINS-I V2 is summarized in **Supplementary Table 1** . Among the five included studies, two were judged to have low overall risk of bias, whereas three were considered moderate risk of bias. Across studies, bias due to confounding and bias in selection of participants were the most frequent concerns, reflecting the retrospective design and lack of blinding. Bias due to outcome measurement was generally low, as all studies relied on the same definitions, despite the retrospective data collection. Other domains, including classification of interventions, deviations from intended interventions, missing data, and selective reporting, were mostly judged as low to moderate risk. The detailed electronic search strategy for each database is provided in **Supplementary Table 2** .

**Table:** **Table 2** Pooled estimates with heterogeneity.

	Relative risk (95% confidence interval)	*P* value	Heterogeneity	Egger’s test
Recurrent biliary obstruction EUS-HGS+AS (5 studies, 217 patients) EUS-HGS (5 studies, 338 patients)	0.30 [0.18–0.49]	<0.001	I ^2^ 0.0%	0.57
Technical success rate EUS-HGS+AS (5 studies, 217 patients) EUS-HGS (5 studies, 338 patients)	0.94 [0.85–1.05]	0.27	I ^2^ 80.9%	0.002
Clinical success rate EUS-HGS+AS (5 studies, 217 patients) EUS-HGS (5 studies, 338 patients)	1.02 [0.94–1.11]	0.57	I ^2^ 64.1%	0.97
Adverse event rate EUS-HGS+AS (5 studies, 217 patients) EUS-HGS (5 studies, 338 patients)	0.88 [0.50–1.55]	0.66	I ^2^ 33.6%	0.12
Severe adverse event rate EUS-HGS+AS (4 studies, 186 patients) EUS-HGS (4 studies, 213 patients)	0.26 [0.03–2.22]	0.22	I ^2^ 0.0%	<0.001
	Standard mean difference (95% confidence interval)	*P* value	Heterogeneity	Egger’s test
Time to recurrent biliary obstruction EUS-HGS+AS (4 studies, 186 patients) EUS-HGS (4 studies, 213 patients)	+ 4.02 [0.57–7.47]	0.02	99.0%	0.04
Mean procedure time EUS-HGS+AS (3 studies, 105 patients) EUS-HGS (3 studies, 132 patients)	+0.38 [-0.12–0.87]	0.13	71.8%	0.85
Overall survival EUS-HGS+AS (4 studies, 186 patients) EUS-HGS (4 studies, 213 patients)	0.18 [-0.20–0.52]	0.38	67.9%	0.85
AS, antegrade stenting; EUS-HGS, endoscopic ultrasound guided hepaticogastrostomy.				

### Primary outcome: Pooled recurrent biliary obstruction

Patients who underwent biliary drainage with EUS-HGS combined to AS had a significantly lower risk of RBO; relative risk (RR) 0.30 [95% CI 0.18–0.49]; *P* <0.001, as shown in Fig. 1 with no heterogenicity (I ^2^ 0.0%) ( Table 2 ) *.* Risk reduction for RBO was -19.4% ([95% C.I. -32.5 to -6.3%]; *P* = 0.004). No publication bias was observed, as shown in **Supplementary Fig. 2a** and confirmed by Egger’s test, *P* = 0.57.

**Fig. 2:**
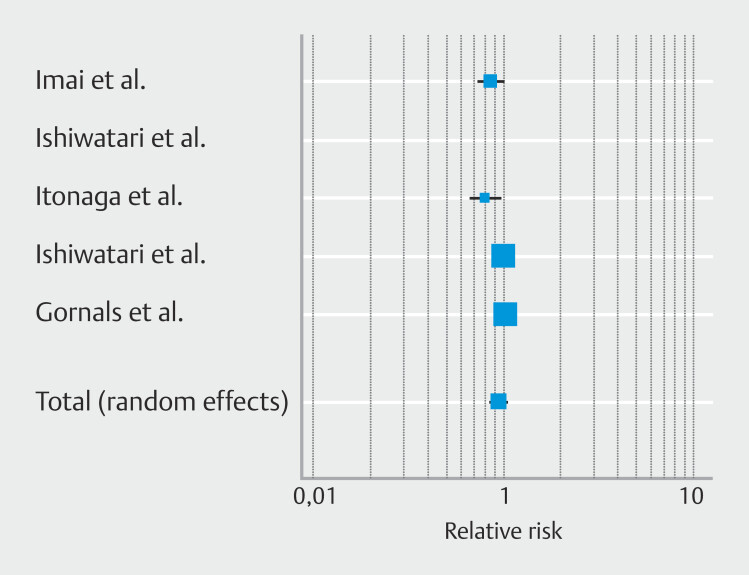
Forest plot reporting pooled estimates for technical success rate.

### Secondary outcomes: Pooled technical success rate

Pooled technical success was similar in the two groups, RR 0.94; [95% CI 0.85–1.05]; *P* = 0.27, as shown in Fig. 2 high heterogeneity (I ^2^ 80.9%) was observed as well as ( Table 2 ) a potential publication bias as shown in **Supplementary Fig. 2b** and confirmed by Egger’s test ( *P* = 0.002).

### Secondary outcomes: Pooled clinical success rate

**Fig. 3:**
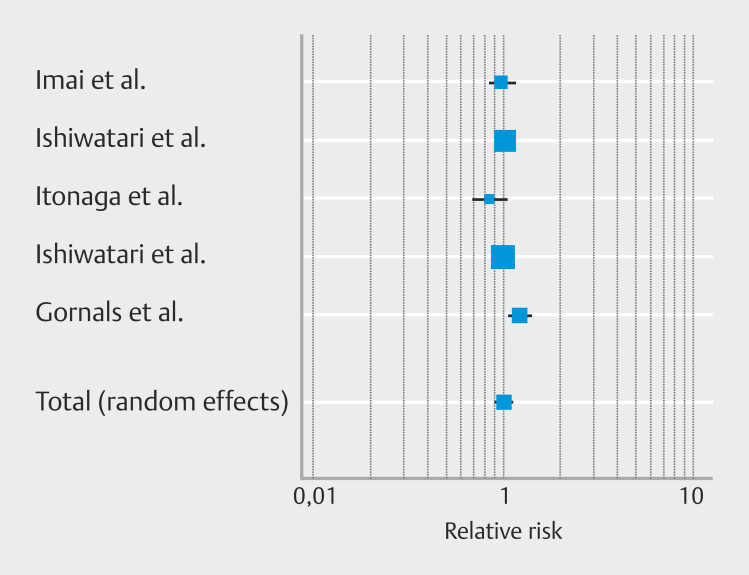
Forest plot reporting pooled incidence of clinical success rate.

Pooled clinical success rate was similar in patients who underwent EUS-HGS plus AS and EUS-HGS alone; RR was 1.02 [95% CI 0.94–1.11]; *P* = 0.57 ( Fig. 3 ); high heterogenicity (I ^2^ 64.1%) was found but no publication bias was observed ( **Supplementary Fig. 2c** ) (Egger’s test *P* = 0.97).

### Secondary outcomes: Safety profile

**Fig. 4:**
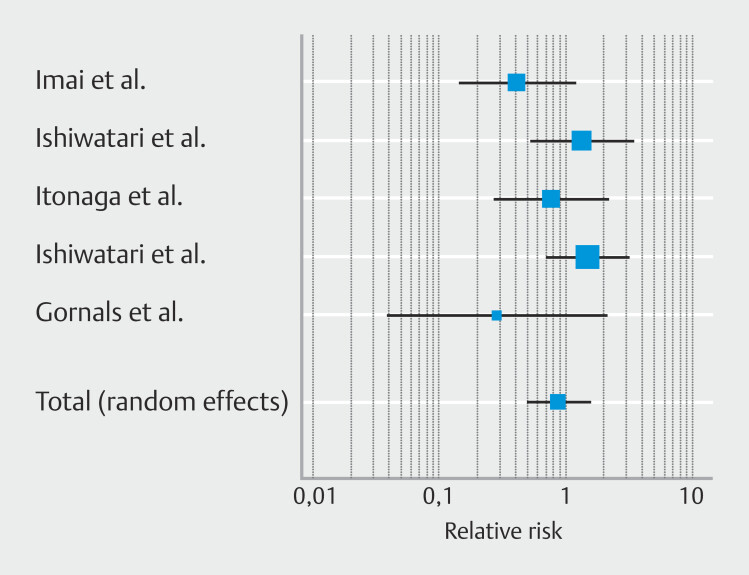
Forest plot reporting pooled incidence of adverse events rate.

Overall pooled incidence of AEs was similar in the two groups; RR was 0.88 [95% CI 0.50–1.55]; *P* = 0.66 ( Fig. 4 ); moderate heterogenicity (I ^2^ 33.6%) was observed with no publication bias ( **Supplementary Fig. 2d** ) was observed (Egger’s test *P* = 0.12). Pooled incidence of severe AEs was comparable in EUS-HGS+AG and EUS-HGS alone; RR 0.26 [95% CI 0.03–2.22]; *P* = 0.22, with low level of heterogenicity (I ^2^ 0%) ( Table 2 ). Potential publication bias was observed and confirmed by Egger’s test ( *P* < 0.001). Pooled incidence of AEs according to severity was reported in **Supplementary Fig. 3a, Supplementary Fig. 3b, and Supplementary Fig. 3c.**

### Secondary outcomes: Time to recurrent biliary obstruction

**Fig. 5:**
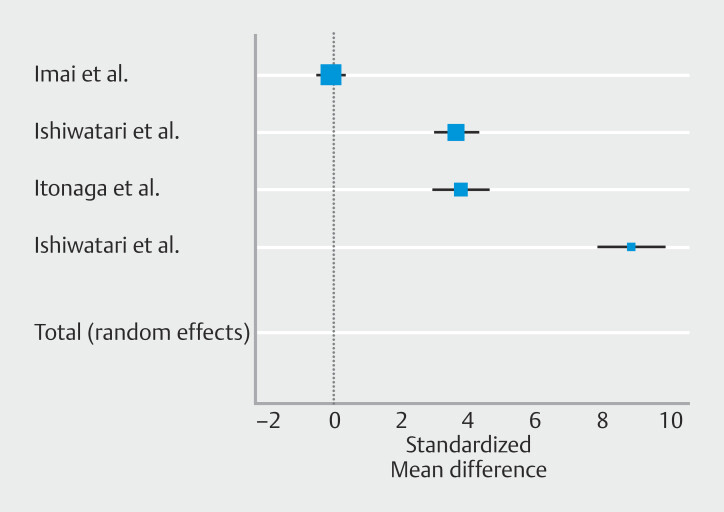
Forest plot reporting pooled incidence for time to recurrent biliary obstruction.

Patients who underwent biliary drainage through EUS-GHS combined with AS showed significantly longer time to RBO ( Fig. 5 ); standardized mean difference of time to RBO was +4.02 [95% CI 0.57–7.47]; *P* = 0.02; high level of heterogenicity (I ^2^ 99.0%) together with a potential publication bias were observed Egger’s test ( *P* = 0.04).

### Secondary outcomes: Procedure time

Mean procedure time was similar among the two groups ( **Supplementary Fig. 4** ). Standardized mean difference was +0.38 [95% CI -0.12–0.87]; *P* = 0.13; high level of heterogeneity was found (I ^2^ = 71.8%) with no publication bias (Egger’s test *P* = 0.85).

### Secondary outcomes: Overall survival

Overall survival was similar in EUS-HGS+AG and EUS-HGS alone groups; standardized mean difference +0.18 [95% CI -0.20–0.52]; P = 0.38, ( **Supplementary Fig. 5** ), with moderate level of heterogenicity (I ^2^ 67.9%) ( Table 2 ). No publication bias was observed (Egger’s test P = 0.85).

### Sensitivity analysis for primary outcome

Sensitivity analysis was shown in Table 3 .

**Table:** **Table 3** Sensitivity analysis for primary outcome (recurrent biliary obstruction).

	Relative Risk (95% confidence interval)	*P* value	Heterogeneity
Study population			
< 80 patients (2 studies; 141 patients)	0.27 [0.13–0.53]	< 0.001	I ^2^ 0.0%
> 80 patients (3 studies; 414 patients)	0.31 [0.11–0.86]	0.02	I ^2^ 40.1%
Study design			
Single center (3 studies; 237 patients)	0.34 [0.19–0.58]	< 0.001	I ^2^ 0.0%
Multicenter (2 studies; 318 patients)	0.18 [0.05–0.69]	0.01	I ^2^ 18.2%
Study design			
Retrospective (3 studies; 237 patients)	0.34 [0.19–0.58]	< 0.001	I ^2^ 0.0%
Propensity score matched (2 studies; 318 patients)	0.18 [0.05–0.69]	0.01	I ^2^ 18.2%
Publication year			
Before 2023 (2 studies; 175 patients)	0.34 [0.14 –0.85]	0.02	I ^2^ 36.1%
After 2023 (3 studies; 380 patients)	0.26 [0.13–0.51]	< 0.001	I ^2^ 0.0%
Type of stents for antegrade stenting			
Metal stents (4 studies; 493 patients)	0.28 [0.14–0.57]	0.001	I ^2^ 22.2%
Plastic stents (1 study; 62 patients)	N/A	N/A	N/A
Location of malignant biliary obstruction			
Only distal (2 studies; 258 patients)	0.26 [0.06–1.21]	0.09	I ^2^ 69.6%
Proximal and distal (3 studies; 297 patients)	0.28 [0.15–0.54]	< 0.001	I ^2^ 0.0%
N/A, not applicable.			

The results showed that most heterogeneity was observed in large studies (> 80 patients) (I ^2^ 40.1%) and in studies enrolling only patients with distal MBO (I ^2^ 69.6%). Mild heterogeneity (I ^2^ 22.2%) was found in the four studies in which only metal stents were used for AS.

## Discussion

Results of this study showed that AS combined with EUS-HGS reduces incidence of RBO in patients with MBO, without impairing any other clinical outcome, such as technical and clinical success rates or incidence of AEs. Moreover, time to RBO appears significantly longer in patients who received both EUS-HGS and AS compared to EUS-HGS alone.

This meta-analysis included five comparative studies, reporting performance of EUS-HGS combined with AS versus EUS-HGS alone in 555 patients suffering from MBO. The quantitative analysis showed a significantly lower incidence of RBO in the EUS-HGS+AS group (relative risk 0.30; risk reduction -19.4%) with no difference in terms of technical success (RR 0.94), clinical success (RR 1.02), incidence of AEs (RR 0.88) and severe AEs (RR 0.26). Neither the technical success rate nor the mean procedure time were different among the two groups. All studies included in this meta-analysis had a retrospective design, which inherently limits causal inference and increases risk of selection bias, information bias, and unmeasured confounding. In detail, treatment allocation was not randomized, and important clinical or procedural factors influencing the choice to perform AS may not have been fully captured. As a result, the certainty of evidence remains limited, and pooled estimates should be interpreted with caution. These findings, therefore, should be considered hypothesis-generating and underscore the need for well-designed prospective and randomized controlled trials (RCTs) to definitively clarify the role of AS during EUS-guided hepaticogastrostomy.

The combination of EUS-HGS with AS was demonstrated to significantly reduce incidence of RBO and increase time to RBO, leading to better patient prognosis. A previous study showed that EUS-HGS was effective in perihilar cholangiocarcinoma leading to improvement in jaundice and patient survival 20 .

Interestingly, we observed a trend toward reduction in incidence of severe AEs in the EUS-HGS+AS group; we speculate that concomitant antegrade and transgastric drainage could reduce risk of bile leak compared with EUS-HGS alone.

The lower RBO in the EUS-HGS+AS can be attributed to the fact that when one stent becomes occluded, the other stent offers an alternative outlet 10 13 . A previous study reported stent patency in EUS-HGS of 3 to 6 months 21 , whereas stent patency in studies using ≥ 10 mm EUS-HGS stent was approximately 6.3 months 22 23 . On the other hand, a recent study by Inoue et al. reported a median time to RBO of 8 months in 57 patients with MBO who underwent EUS-HGS+AS ( Table 4 ) 24 .

**Table:** **Table 4** Cause of EUS-hepaticogastrostomy or antegrade stent obstruction.

	EUS-HGS+AS	EUS-HGS alone
Gornals et al. 2025	Not reported	Not reported
Ishiwatari et al. 2024	Not reported	Not reported
Itonaga et al. 2024	Cancer invasion at AS stent (4 patients)	Food impaction (10 patients) Hyperplasia into EUS-HGS stent (7 patients)
Ishiwatari et al. 2022	Hyperplasia into EUS-HGS stent (3 patients) Sludge (1 patient) Unknown (1 patient) Cancer invasion at AS stent (4 patients)	Hyperplasia into EUS-HGS stent (8 patients) Sludge (4 patients) EUS-HGS stent migration (1 patient) Biliary stricture developing into the hilar part (1 patient) EUS-HGS stent migration into the Esophagus (1 patient)
Imai et al. 2017	Not reported	Not reported
AS, antegrade stenting; EUS-HGS, endoscopic ultrasound hepaticogastrostomy.		

RBO is mostly caused by food impaction or sludge (21.1%) and tissue hyperplasia at the level of the uncovered portion of the stent (15.8%) 25 . In this meta-analysis, data on the cause of RBO were reported in only two studies 15 17 . Incidence of RBO due to food impaction or sludge and tissue hyperplasia seems reduced in the EUS-HGS+AS combination group compared with EUS-HGS alone. Again, we speculate that two drainage routes could overcome the limitation of each route alone, thus decreasing incidence of RBO.

Analysis of time to RBO was characterized by extremely high heterogeneity (I² = 99%), reflecting substantial clinical and methodological variability among the included studies. Potential sources of heterogeneity include differences in stent materials, puncture site selection (segment II versus segment III), variations in AS techniques, and heterogeneity in patient populations. Although a pooled estimate was calculated using a random-effects model, this result should be interpreted with caution and regarded as exploratory. Robust conclusions regarding time to RBO will require prospective studies with standardized procedural approaches and more homogeneous reporting.

We observed slightly higher technical success in the EUS-HGS alone group probably because it requires less device exchanges and manipulations than EUS-HGS+AS. A recent systematic review of non-comparative studies also demonstrated a technical success rate of 94.4% and 89.7% for EUS-HGS alone and EUS-HGS+AS, respectively 6 .

Overall incidence of AEs was similar between the two groups ( Table 5 ) *.* However, in the AE analysis, post-procedure pancreatitis occurred more frequently after EUS-HGS+AS, plausibly related to mechanical compression of the pancreatic orifice by the transpapillary stent 26 . In our pooled data, pancreatitis was observed in 14 of 186 patients (7.5%) with EUS-HGS+AS versus one of 213 (0.47%) with EUS-HGS, yielding a RR of 16.0 ( *P* = 0.0002). Bile leak represents one of the most frequent and life-threatening AEs after EUS-HGS 4 27 . Relative incidence of bile leak was reduced in the EUS-HGS+AS group because reduced pressure in the biliary tree through the two drainage routes seems to protect from risk of leak 14 . Consequently, relative incidence of severe biliary peritonitis was lower in the EUS-HGS+AS group compared with EUS-HGS alone (3% vs. 12.5%, respectively).

**Table:** **Table 5** Detailed description of adverse events.

	EUS-HGS+AS	EUS-HGS alone
Overall AEs	34	54
Gornals et al. 2025	Not reported	Not reported
Ishiwatari et al. 2024	Mild Peritonitis (7 patients), bleeding (1 patient), cholangitis (1 patient), pancreatitis (3 patients) Moderate Bleeding (2 patients), cholangitis (1 patient), sepsis (1 patient), pancreatitis (1 patient)	Mild Peritonitis (3 patients), cholangitis (1 patient), pancreatitis (1 patient), Moderate Peritonitis (1 patient), migration of EUS-HGS stent (1 patient), cholangitis (2 patients), biloma (1 patient) Severe Peritonitis (1 patient), migration of EUS-HGS stent (1 patient), bleeding (1 patient), sepsis (1 patient),
Itonaga et al. 2024	Mild Pancreatitis (3 patients), cholangitis (1 patient) Moderate Bile leak (1 patient),	Mild Bile leak (2 patients), aspiration pneumonia (1 patient), bleeding (1 patient) Moderate Bile leak (2 patients), abdominal pain (1 patient)
Ishiwatari et al. 2022	Mild Pancreatitis (5 patients) Moderate Bleeding (2 patients)	Mild Peritonitis (4 patients), cholangitis (2 patients) Moderate Peritonitis (1 patient) Fatal Sepsis (1 patient)
Imai et al. 2017	Mild Pancreatitis (2 patients), bile leak (1 patient), cholangitis (1 patient)	Mild Bile leak (7 patients), cholangitis (2 patients) Moderate Stent migration (2 patients)
AS, antegrade stenting; EUS-HGS, endoscopic ultrasound hepaticogastrostomy.		

We acknowledge that two meta-analyses recently have been published in the same field 12 13 . However, this study also included recently published international large PSM studies, corroborating the previously observed pooled outcomes; moreover, this study included only comparative studies, limiting potential selection bias.

In addition to the studies included in the present meta-analysis, two recent comparative investigations deserve consideration. Zhang et al. 28 compared EUS-HGS with AS versus EUS-HGS alone after failed ERCP in a similar setting and reported higher biliary drainage effectiveness and longer time to stent dysfunction or death in the combined approach. However, we decided not to include this study in the quantitative synthesis because data on RBO are lacking, as defined in the present analysis as the main outcome measure, and AS was in some cases performed in a separate session from EUS-HGS, potentially introducing procedural heterogeneity and outcome reporting bias.

Interestingly, Takenaka et al. 29 reported that EUS-guided hepaticogastrostomy combined with AS was associated with significantly higher radiation exposure compared with EUS-HGS alone, reflecting need for prolonged fluoroscopy during guidewire manipulation across the biliary stricture and transpapillary stent deployment. This finding is clinically relevant, particularly in high-volume centers and in patients requiring repeated interventions, because cumulative radiation exposure may affect both patients and endoscopy staff. Although our meta-analysis did not identify a significant difference in overall procedure time between the two approaches, fluoroscopy duration and radiation dose were not consistently reported across the included studies and could not be quantitatively analyzed. Therefore, the potential increase in radiation exposure with EUS-HGS combined with AS should be balanced against its demonstrated benefit in reducing RBO and prolonging stent patency. Future prospective studies should systematically report fluoroscopy time and radiation dose to better define the risk-benefit profile of combined antegrade and transmural drainage strategies.

Our meta-analysis presents several limitations. First, all retrospective studies could have a potential selection bias with subsequent overestimation of reported outcomes. Second, most studies have been conducted in tertiary-referral centers from highly experienced operators; therefore, performance of EUS-HGS and AS cannot be necessarily translated to other centers. The third limitation is heterogeneity of the included patient populations, particularly regarding the site of MBO. Although most studies focused on distal MBO, some included proximal cases, which could influence procedure outcomes and complication rates. We performed sensitivity analyses stratified by distal versus proximal obstruction to assess impact of this variability; these analyses suggest that the overall conclusions remain broadly consistent, although some differences in specific outcomes were observed. This heterogeneity should be considered when interpreting the results and applying them to clinical practice. Moreover, two studies with the same first author, namely Ishiwatari et al., have been included in the quantitative analysis 15 16 . Because any potential overlap between the two study populations represents a major limitation due to a potential “double-counting bias,” we excluded possible population overlap through contacting the corresponding authors. Finally, inclusion of both studies was deemed appropriate given the distinct populations and analytical frameworks, particularly use of PSM in the latter study. Nevertheless, a potential bias cannot be completely excluded and may have influenced pooled estimates. Finally, in the study by Itonaga et al. 17 , six patients were excluded from the EUS-HGS+AS secondary to failed antegrade stenting because the guidewire could not pass through the biliary stricture site. On the other hand, in the study by Ishiwatari et al. 2024 16 , and Imai et al. 18 in the EUS-HGS+AG group, insertion of a stent beyond the biliary obstruction failed in three and six patients, respectively; however, they were considered and analyzed as technical failures, and in the study by Ishiwatari et al. in 2022 15 , all cases of EUS-HGS+AG were successful. Therefore, potential bias from groups crossover seems to be marginal and negligible. On the other hand, inclusion of comparative studies, with well-defined and homogeneous inclusion criteria together with the low heterogeneity of study designs, underscores strengths of this meta-analysis. Another important limitation relates to heterogeneity in stent materials used across studies. Although some cohorts exclusively employed self-expandable metal stents for EUS-HGS and AS, others included a mixture of metal and plastic stents. Given the known impact of stent material on patency, this heterogeneity represents a potential source of residual confounding, particularly for time to RBO outcomes. Owing to the limited number of studies and insufficient reporting of stent-specific data, formal subgroup or meta-regression analyses were not feasible. Consequently, influence of stent material on pooled estimates cannot be fully disentangled. Absence of prospective registration represents a methodological limitation. Most studies included in this meta-analysis were conducted at high-volume, expert centers, often within single-center or specialized referral settings. In addition, study designs ranged from unadjusted retrospective cohorts to PSM analyses, introducing variability in methodological rigor. Outcomes achieved in such highly specialized environments may not be readily generalizable to lower-volume centers or community practice, where procedural expertise, patient selection, and peri-procedural management may differ. These factors should be considered when applying the present findings to broader clinical contexts.

## Conclusions

In conclusion, EUS-HGS+AS reduces incidence of RBO and increases time to RBO compared with EUS-HGS alone, with no difference in terms of technical and clinical success and incidence of AEs. Based on the improving outcomes and survivals observed in patients with MBO receiving chemotherapy, identification of a biliary drainage strategy able to reduce need for biliary reinterventions could dramatically impact patient outcomes, chemotherapy continuation, and quality of life. On this basis, prospective RCTs are warranted to confirm these results.

## Supplementary Material

Supplementary Material Zusatzmaterial

## References

[1] Okusaka T Nakamura M Yoshida M Clinical Practice Guidelines for Pancreatic Cancer 2022 from the Japan Pancreas Society: a synopsis Int J Clin Oncol 2023 28 493 511 36920680 10.1007/s10147-023-02317-x PMC10066137

[2] Dumonceau JM Tringali A Papanikolaou IS Endoscopic biliary stenting: indications, choice of stents, and results: European Society of Gastrointestinal Endoscopy (ESGE) Clinical Guideline - Updated October 2017 Endoscopy 2018 50 910 930 10.1055/a-0659-9864 30086596

[3] Marzioni M Crino SF Lisotti A Biliary drainage in patients with malignant distal biliary obstruction: results of an Italian consensus conference Surg Endosc 2024 38 6207 6226 39317905 10.1007/s00464-024-11245-4 PMC11525304

[4] van der Merwe SW van Wanrooij RLJ Bronswijk M Therapeutic endoscopic ultrasound: European Society of Gastrointestinal Endoscopy (ESGE) Guideline Endoscopy 2022 54 185 205 10.1055/a-1717-1391 34937098

[5] Paik WH Park DH Outcomes and limitations: EUS-guided hepaticogastrostomy Endosc Ultrasound 2019 8 S44 S49 10.4103/eus.eus_51_19 31897379 PMC6896431

[6] Moond V Loganathan P Koyani B Efficacy and safety of EUS-guided hepatogastrostomy: A systematic review and meta-analysis Endosc Ultrasound 2024 13 171 182 10.1097/eus.0000000000000055 39318645 PMC11419430

[7] Conroy T Desseigne F Ychou M FOLFIRINOX versus gemcitabine for metastatic pancreatic cancer N Engl J Med 2011 364 1817 1825 10.1056/NEJMoa1011923 21561347

[8] Golan T Hammel P Reni M Maintenance olaparib for germline BRCA-mutated metastatic pancreatic cancer N Engl J Med 2019 381 317 327 10.1056/NEJMoa1903387 31157963 PMC6810605

[9] Binda C Dajti E Giuffrida P Efficacy and safety of endoscopic ultrasound-guided hepaticogastrostomy: a meta-regression analysis Endoscopy 2024 56 694 705 10.1055/a-2282-3350 38447958

[10] Ogura T Kitano M Takenaka M Multicenter prospective evaluation study of endoscopic ultrasound-guided hepaticogastrostomy combined with antegrade stenting (with video) Dig Endosc 2018 30 252 259 29055054 10.1111/den.12976

[11] Yamamoto K Itoi T Tsuchiya T EUS-guided antegrade metal stenting with hepaticoenterostomy using a dedicated plastic stent with a review of the literature (with video) Endosc Ultrasound 2018 7 404 412 30531024 10.4103/eus.eus_51_18 PMC6289013

[12] Martins OC Antunes V da Costa MCP Evaluating the use of EUS-guided hepaticogastrostomy combined with antegrade stenting for malignant biliary obstruction and comparing to EUS-guided hepaticogastrostomy alone for patients who failed ERCP: a pairwise and single-arm meta-analysis Surg Endosc 2025 39 3786 3796 40325242 10.1007/s00464-025-11760-y

[13] Paraskevopoulos P Obeidat M Bednárik D Hepaticogastrostomy versus hepaticogastrostomy with antegrade stenting for malignant biliary obstruction: a systematic review and meta-analysis Therap Adv Gastroenterol 2024 17 17562848241273085 10.1177/17562848241273085 PMC11500218 39449980

[14] Page MJ McKenzie JE Bossuyt PM The PRISMA 2020 statement: an updated guideline for reporting systematic reviews BMJ 2021 372 n71 10.1136/bmj.n71 33782057 PMC8005924

[15] Ishiwatari H Ishikawa K Niiya F Endoscopic ultrasound-guided hepaticogastrostomy versus hepaticogastrostomy with antegrade stenting for malignant distal biliary obstruction J Hepatobiliary Pancreat Sci 2022 29 703 712 35094496 10.1002/jhbp.1118

[16] Ishiwatari H Ogura T Hijioka S et al A EUS-guided hepaticogastrostomy versus EUS-guided hepaticogastrostomy with antegrade stent placement in patients with unresectable malignant distal biliary obstruction: a propensity score-matched case-control study Gastrointest Endosc 2024 100 66 75 38382887 10.1016/j.gie.2024.02.012

[17] Itonaga M Ashida R Hatamaru K Endoscopic ultrasound-guided hepaticogastrostomy vs. antegrade metal stent placement keeping an access route in patients with malignant biliary obstruction Int J Clin Oncol 2024 29 1500 1508 38972023 10.1007/s10147-024-02584-2

[18] Imai H Takenaka M Omoto S Utility of endoscopic ultrasound-guided hepaticogastrostomy with antegrade stenting for malignant biliary obstruction after failed endoscopic retrograde cholangiopancreatography Oncology 2017 93 69 75 29258066 10.1159/000481233

[19] Gornals JB Samanta J Aparicio Tomo JR Endoscopic Ultrasound-guided hepaticogastrostomy versus hepaticogastrostomy with antegrade stenting for malignant biliary obstruction: a multicentre propensity score analysis Endoscopy 2025 57 S76 S77

[20] Schoch A Lisotti A Walter T Efficacy of EUS-guided hepaticogastrostomy in prolonging survival of patients with perihilar cholangiocarcinoma Endosc Ultrasound 2022 11 487 494 36537386 10.4103/EUS-D-22-00014 PMC9921975

[21] Teoh AYB Dhir V Kida M Consensus guidelines on the optimal management in interventional EUS procedures: results from the Asian EUS group RAND/UCLA expert panel Gut 2018 67 1209 1228 29463614 10.1136/gutjnl-2017-314341

[22] Nakai Y Sato T Hakuta R Long-term outcomes of a long, partially covered metal stent for EUS-guided hepaticogastrostomy in patients with malignant biliary obstruction (with video) Gastrointest Endosc 2020 92 623 631 32278705 10.1016/j.gie.2020.03.3856

[23] Nakai Y Isayama H Yamamoto N Safety and effectiveness of a long, partially covered metal stent for endoscopic ultrasound-guided hepaticogastrostomy in patients with malignant biliary obstruction Endoscopy 2016 48 1125 1128 27716860 10.1055/s-0042-116595

[24] Inoue T Kitano R Ibusuki M Endoscopic ultrasound-guided hepaticogastrostomy with antegrade stenting without dilation device application for malignant distal biliary obstruction in pancreatic cancer Dig Dis Sci 2023 68 2090 2098 36350476 10.1007/s10620-022-07749-5

[25] Hedjoudje A Pokossy Epee J Long-term outcomes of endoscopic ultrasound-guided hepaticogastrostomy in patients with malignant biliary obstruction United European Gastroenterol J 2024 12 1044 1055 10.1002/ueg2.12552 PMC11485310 38578654

[26] Ogura T Masuda D Imoto A EUS-guided hepaticogastrostomy combined with fine-gauge antegrade stenting: a pilot study Endoscopy 2014 46 416 421 24573771 10.1055/s-0034-1365020

[27] Isayama H Nakai Y Itoi T Clinical practice guidelines for safe performance of endoscopic ultrasound/ultrasonography-guided biliary drainage: 2018 J Hepatobiliary Pancreat Sci 2019 26 249 269 10.1002/jhbp.631 31025816 PMC7064894

[28] Zhang Y Wang X Sun K Application of endoscopic ultrasound-guided hepaticogastrostomy combined with antegrade stenting in patients with malignant biliary obstruction after failed ERCP Surg Endosc 2022 36 5930 5937 35178592 10.1007/s00464-022-09117-w

[29] Takenaka M Rehani MM Hosono M Comparison of radiation exposure between endoscopic ultrasound-guided hepaticogastrostomy and hepaticogastrostomy with antegrade stenting J Clin Med 2022 11 1705 35330030 10.3390/jcm11061705 PMC8951780

