# Supplementary material for: EUS-HGS with antegrade stenting vs. hepaticogastrostomy alone for malignant biliary drainage: Systematic review and meta-analysis
Source: Endosc Int Open. 2026 Apr 10;14:a28406762. doi: 10.1055/a-2840-6762 (PMC13077230; doi:10.1055/a-2840-6762)

Supplementary Table 1 Quality assessment of the included studies according to the Risk Of Bias In Non-randomized Studies – of Interventions (ROBINS-I) tool, Version 2.

	Confounding	Selection of participants	Classification of intervention	Deviation from intended intervention	Missing data	Outcome measurement	Selection of reported result	Overall quality
Imai et al. 2017	Moderate	Moderate	Low	Low	Low	Low	Low	Moderate
Ishiwatari et al. 2022	Moderate	Moderate	Low	Low	Low	Low	Low	Moderate
Itonaga et al. 2024	Low	Moderate	Low	Low	Low	Low	Low	Low
Ishiwatari et al. 2024	Low	Moderate	Low	Low	Low	Low	Low	Low
Gornals et al. 2024	Moderate	Moderate	Low	Low	Moderate	Low	Moderate	Moderate

Available at https://www.riskofbias.info/welcome/robins-i-v2

Supplementary Table 2 Detailed search strategy for all databases.

Database	Search strategy	Date of last search	Records retrieved (n)
PubMed/ MEDLINE	("Endoscopic Ultrasound"[Mesh] OR "endoscopic ultrasound"[tiab] OR EUS[tiab]) AND ("Hepaticogastrostomy"[tiab] OR "hepatic gastro-stomy"[tiab] OR EUS-HGS[tiab])) AND (("Antegrade stenting"[tiab] OR "antegrade drainage"[tiab] OR "antegrade biliary stenting"[tiab]) OR ("stent"[tiab] OR "biliary stent"[tiab])) AND (("Malignant biliary obstruction"[tiab] OR "malignant biliary stricture"[tiab] OR "biliary obstruction"[tiab] OR cholangiocarcinoma[tiab] OR pancreatic cancer[tiab])	June 30, 2025	141
Embase	('endoscopic ultrasound'/exp OR 'endoscopic ultrasound':ti,ab OR EUS:ti,ab) AND ('hepaticogastrostomy':ti,ab OR 'eus guided hepaticogastrostomy':ti,ab OR EUS-HGS:ti,ab) AND ('antegrade stenting':ti,ab OR 'antegrade biliary drainage':ti,ab OR 'biliary stent'/exp OR 'biliary stent':ti,ab) AND ('malignant biliary obstruction':ti,ab OR 'biliary tract cancer'/exp OR 'cholangiocarcinoma'/exp OR 'pancreatic cancer'/exp)	June 30, 2025	48
Scopus	(TITLE-ABS-KEY("endoscopic ultrasound") OR TITLE-ABS-KEY(EUS)) AND (TITLE-ABS-KEY("hepaticogastrostomy") OR TITLE-ABS-KEY("hepatic gastrostomy") OR TITLE-ABS-KEY(EUS-HGS)) AND ((TITLE-ABS-KEY("antegrade stenting") OR TITLE-ABS-KEY("antegrade drainage") OR TITLE-ABS-KEY("antegrade biliary stenting")) OR (TITLE-ABS-KEY(stent) OR TITLE-ABS-KEY("biliary stent"))) AND (TITLE-ABS-KEY("malignant biliary obstruction") OR TITLE-ABS-KEY("malignant biliary stricture") OR TITLE-ABS-KEY("biliary obstruction") OR TITLE-ABS-KEY(cholangiocarcinoma) OR TITLE-ABS-KEY("pancreatic cancer"))	June 30, 2025	115
Cochrane Library	("endoscopic ultrasound":ti,ab,kw OR EUS:ti,ab,kw) AND ("hepaticogastrostomy":ti,ab,kw OR "hepatic gastro-stomy":ti,ab,kw OR EUS-HGS:ti,ab,kw) AND (("antegrade stenting":ti,ab,kw OR "antegrade drainage":ti,ab,kw OR "antegrade biliary stenting":ti,ab,kw) OR (stent:ti,ab,kw OR "biliary stent":ti,ab,kw)) AND ("malignant biliary obstruction":ti,ab,kw OR "malignant biliary stricture":ti,ab,kw OR "biliary obstruction":ti,ab,kw OR cholangiocarcinoma:ti,ab,kw OR "pancreatic cancer":ti,ab,kw)	June 30, 2025	25

Web of Science	TS=("endoscopic ultrasound" OR EUS) AND TS=("hepaticogastrostomy" OR "hepatic gastro-stomy" OR EUS-HGS) AND TS=("antegrade stenting" OR "antegrade drainage" OR "antegrade biliary stenting" OR stent OR "biliary stent") AND TS=("malignant biliary obstruction" OR "malignant biliary stricture" OR "biliary obstruction" OR cholangiocarcinoma OR "pancreatic cancer")	June 30, 2025	45
ClinicalTrials.gov	("endoscopic ultrasound" OR EUS) AND ("hepaticogastrostomy" OR "hepatic gastro-stomy" OR EUS-HGS) AND ("antegrade stenting" OR "antegrade drainage" OR "antegrade biliary stenting" OR stent OR "biliary stent") AND ("malignant biliary obstruction" OR "malignant biliary stricture" OR "biliary obstruction" OR cholangiocarcinoma OR "pancreatic cancer")	June 30, 2025	9

Supplementary Fig. 1 PRISMA flow diagram of the study [14].

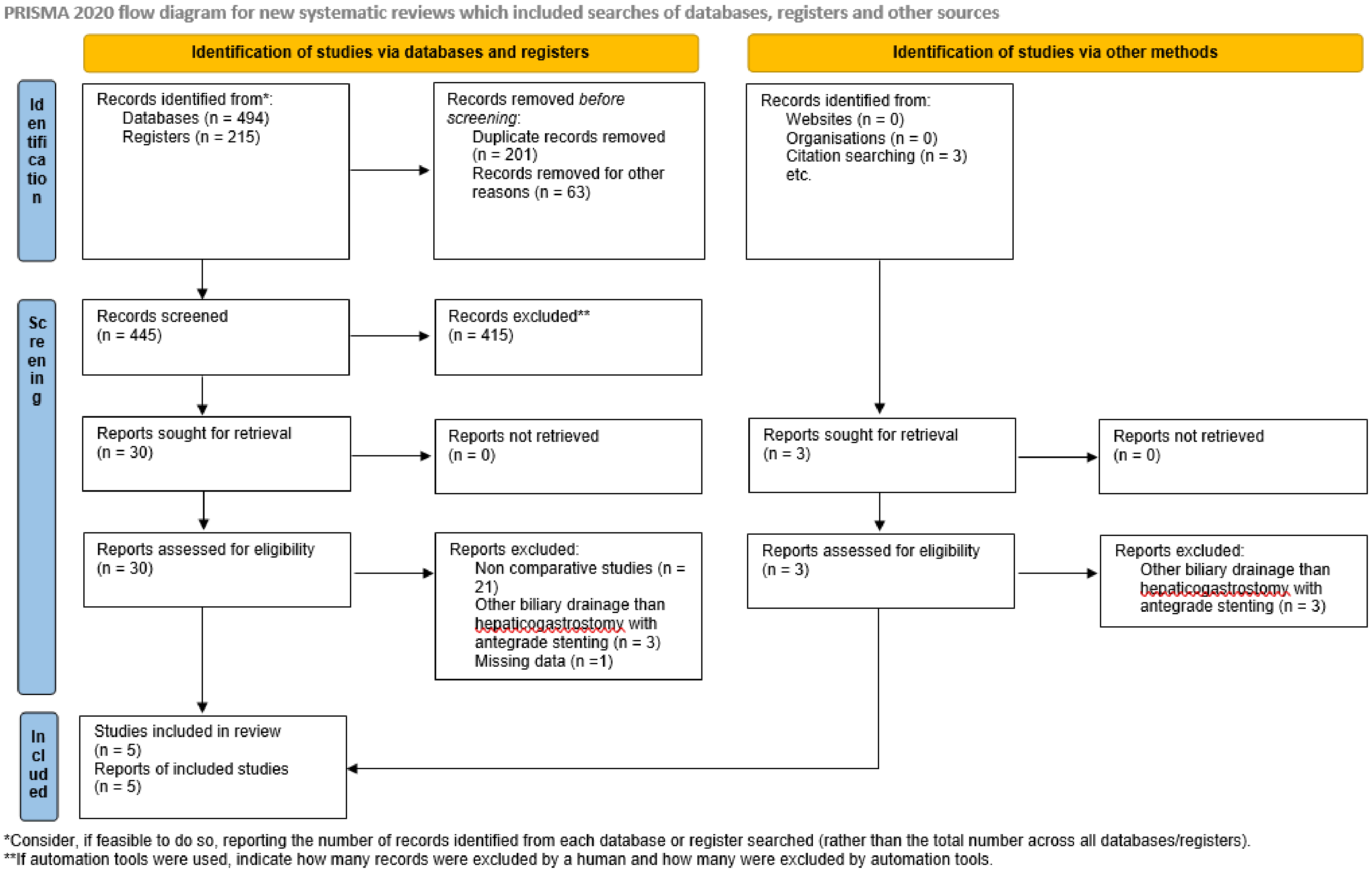

Supplementary Fig. 2 Funnel plots assessment for publication bias. **a** Recurrent biliary obstruction. **b** Technical success rate. **c** Clinical success rate. **d** Adverse events rate.

Supplementary Figure 2a - Funnel plots assessment for publication bias of a) recurrent biliary obstruction

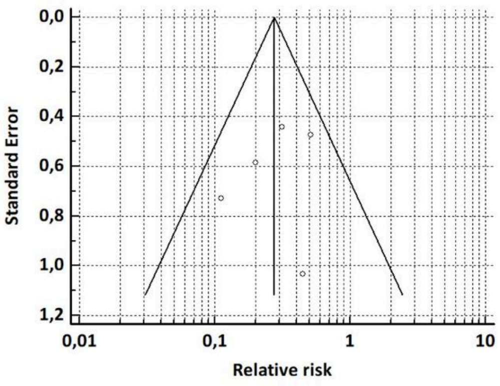

Publication bias

Egger's test	
Intercept	-1,0827
95% CI	-6,5684 to 4,4030
Significance level	P = 0,5745
Begg's test	
Kendall's Tau	-0,2000
Significance level	P = 0,6242

Supplementary Figure 2b - Funnel plots assessment for publication bias of b) technical success rate

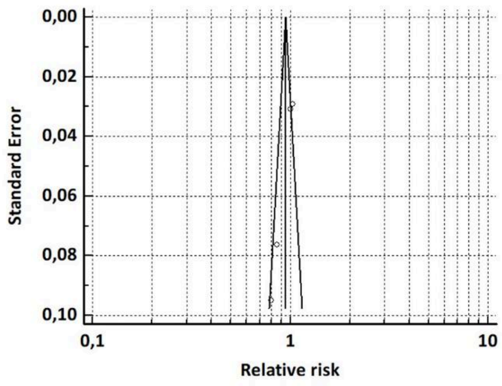

Publication bias

Egger's test	
Intercept	-3,6528
95% CI	-5,5889 to -1,7168
Significance level	P = 0,0148
Begg's test	
Kendall's Tau	-1,0000
Significance level	P = 0,0415

Supplementary Figure 2c - Funnel plots assessment for publication bias of c) clinical success rate

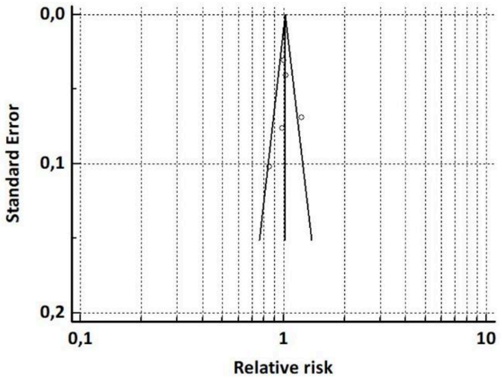

Publication bias

Egger's test	
Intercept	0,07789
95% CI	-6,6832 to 6,8389
Significance level	P = 0,9731
Begg's test	
Kendall's Tau	-0,2000
Significance level	P = 0,6242

Supplementary Figure 2d - Funnel plots assessment for publication bias of d) incidence of adverse event

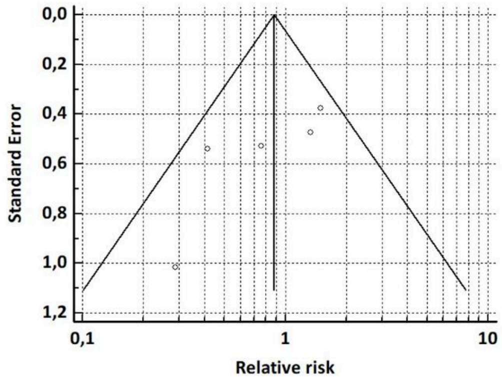

Publication bias

Egger's test	
Intercept	-3,1347
95% CI	-7,6820 to 1,4127
Significance level	P = 0,1158
Begg's test	
Kendall's Tau	-0,8000
Significance level	P = 0,0500

Supplementary Fig. 3 Pooled incidence estimates for adverse events. **a** Mild. **b** Moderate. **c** Severe.

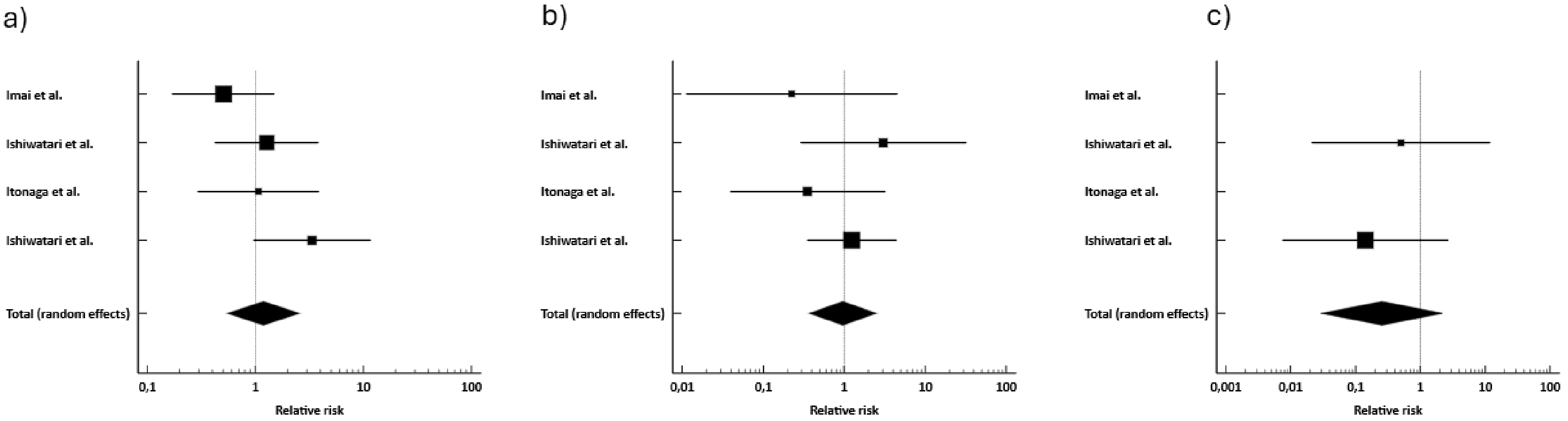

Supplementary Fig. 4 Pooled estimates for mean procedure time.

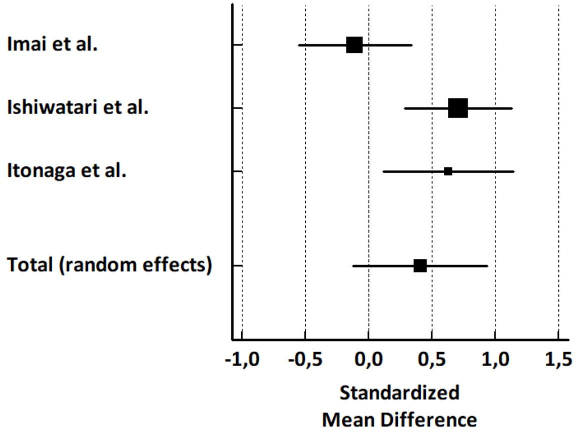

Supplementary Fig. 5 Pooled estimates for overall survival.

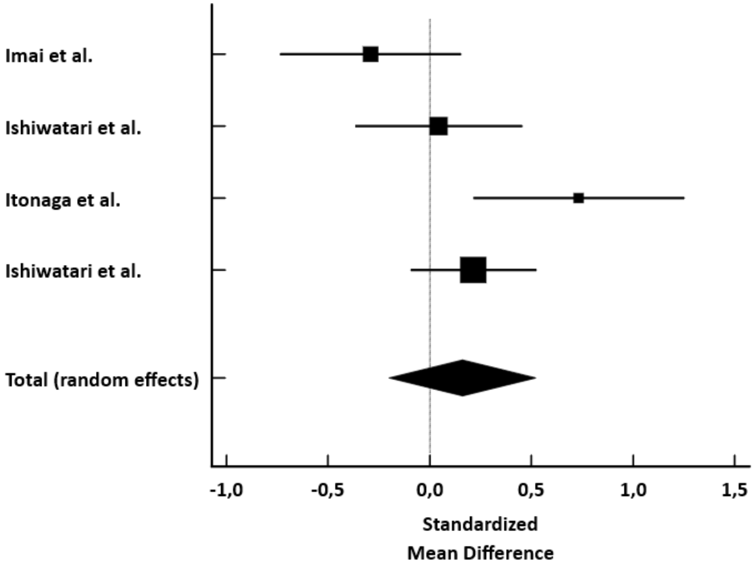

Supplement: Supplementary file 1 — Supplementary Material [file 10-1055-a-2840-6762_28470813.pdf]
